# Comparative Transcriptomics to Identify RNA Writers and Erasers in Microalgae

**DOI:** 10.3390/ijms25158005

**Published:** 2024-07-23

**Authors:** Luca Ambrosino, Alessia Riccardi, Melina S. Welling, Chiara Lauritano

**Affiliations:** 1 Research Infrastructure for Marine Biological Resources Department, Stazione Zoologica Anton Dohrn, Via Acton 55, 80133 Napoli, Italy; luca.ambrosino@szn.it; 2 Department of Integrative Marine Ecology, Stazione Zoologica Anton Dohrn, Villa Comunale, 80121 Napoli, Italy; alessia.riccardi@szn.it; 3 Marine Biology Research Group, Ghent University, Krijgslaan 281, B-9000 Gent, Belgium; melinawelling@gmail.com; 4 Ecosustainable Marine Biotechnology Department, Stazione Zoologica Anton Dohrn, Via Acton 55, 80133 Napoli, Italy

**Keywords:** epitranscriptomics, microalgae, writers, erasers, stress responses

## Abstract

Epitranscriptomics is considered as a new regulatory step in eukaryotes for developmental processes and stress responses. The aim of this study was, for the first time, to identify RNA methyltransferase (writers) and demethylase (erasers) in four investigated species, i.e., the dinoflagellates *Alexandrium tamutum* and *Amphidinium carterae*, the diatom *Cylindrotheca closterium*, and the green alga *Tetraselmis suecica*. As query sequences for the enzymatic classes of interest, we selected those ones that were previously detected in marine plants, evaluating their expression upon nutrient starvation stress exposure. The hypothesis was that upon stress exposure, the activation/deactivation of specific writers and erasers may occur. In microalgae, we found almost all plant writers and erasers (ALKBH9B, ALKBH10B, MTB, and FIP37), except for three writers (MTA, VIRILIZER, and HAKAI). A sequence similarity search by scanning the corresponding genomes confirmed their presence. Thus, we concluded that the three writer sequences were lacking from the studied transcriptomes probably because they were not expressed in those experimental conditions, rather than a real lack of these genes from their genomes. This study showed that some of them were expressed only in specific culturing conditions. We also investigated their expression in other culturing conditions (i.e., nitrogen depletion, phosphate depletion, and Zinc addition at two different concentrations) in *A. carterae*, giving new insights into their possible roles in regulating gene expression upon stress.

## 1. Introduction

Epitranscriptomics has been considered as a new regulatory frontier in eukaryotes for developmental processes and stress responses. More than 150 RNA modifications have been found as post-transcription regulatory marks [1]. The advance of the *-omic* era and available sequencing resources have allowed the production of new molecular data and the identification of several post-transcriptional modifications in various RNAs, such as messenger RNA, ribosomal RNA, transfer RNA, long non-coding RNA, microRNA, and small interfering RNA [2]. RNA alternative splicing, export from the nucleus, alternative polyadenylation, translation, stability, and the storage of mRNA targets have been found to be regulated by specific RNA modifications [3,4,5,6], of which N6-methyladenosine (m6A) is the most frequent [5,7,8,9]. RNA methylation marks are (1) added by proteins known as writers, which are methyltransferases; (2) removed by erasers, which are demethylases; and (3) interpreted by RNA-binding proteins named readers. These modifications have been found in mammals, flies, yeast, and plants [10,11,12,13]. Regarding plants, studies are more frequent in terrestrial plants, especially for the model species *Arabidopsis thaliana*, but also rice and potato, because it was shown that the regulation of m6A levels may induce a direct effect on crop improvement and increase its yields. In animals, members of the multicomponent m6A writer complex include Methyltransferase-Like 3 (METTL3), METTL14, Wilms Tumor 1-Associated Protein (WTAP), KIAA1429, RNA Binding Motif Protein 15 (RBM15), and Zinc finger CCCH domain-containing protein 13 (ZC3H13). In *Arabidopsis*, the ortholog of METTL3 has been found and named MTA, the ortholog of METTL14 has been named MTB, the ortholog of WTAP has been named FIP37, the ortholog of WIRMA has been named VIRILIZER, and, finally, an E3 ubiquitin ligase was recently identified (HAKAI) as part of the multicomponent m6A writer complex. WTAP and WIRMA are mainly heterodimer stabilizers, while METTL3 possesses the main methyltransferase activity. The first eraser was discovered in 2011 [14] (known as the fat mass and obesity-associated protein, or FTO), while in 2013, an α-ketoglutarate-dependent dioxygenase alkB homolog 5 (ALKBH5) [15] was reported. Erasers have been associated with the AlkB RNA demethylase family (ALKBH). ALKBHs have been reported to remove m6A modifications in an α-ketoglutarate- and Fe^2+^-dependent manner.

A recent study that focused on transcriptome-wide N6-methyladenosine (m6A) sequencing in *Arabidopsis thaliana* showed that 33.5% of transcripts had differential m6A levels in different parts of the plant, i.e., leaves, roots, and flowers [16]. Similarly, differences in the transcription levels of m6A writers, erasers, and readers were also found to be associated with different developmental stages, by influencing, for instance, organogenesis, seed development, root and shoot growth, leaf morphology, and fruit ripening [2,17]. In addition to plant parts and developmental stages, epitranscriptomics modifications have been observed to play important roles in plant responses to biotic and abiotic stress exposure. In fact, as recently reviewed by Dhingra et al. [18], it has been suggested that targeting epitranscriptome machinery would help in the engineering of stress tolerance in crops, and would increase their productivity. Gene ontology analyses of high-throughput sequencing in higher plants revealed that m6A modifications were associated with stress-related pathways. Examples of involvement in stress responses in higher plants are low/high temperature, salinity variations, drought, and bacterial attack [19,20,21]. For example, it has been shown that the over-expression of human RNA demethylase in rice induced the up-regulation of pathways related to photosynthesis and nitrogen regulation, increasing crop yields [22]. In addition to terrestrial plants, a recent study by Ruocco and collaborators [23] investigated m6A RNA methylation in relation to biological rhythms in marine plants. They provided the first description of m6A-related genes in seagrasses and studied daily changes in writer and eraser expression levels in *Cymodocea nodosa* and *Zostera marina*. 

To our knowledge, while there are some studies on epigenetics [24,25], not much is currently known regarding epitranscriptomics in marine microalgae. The aim of the current study was (1) to identify, for the first time in microalgae, writers and erasers in four investigated species, i.e., the dinoflagellates *Alexandrium tamutum* and *Amphidinium carterae*, the diatom *Cylindrotheca closterium*, and the green alga *Tetraselmis suecica*, selecting as query sequences for the enzymatic classes of interest those ones that were previously detected in marine angiosperms by Ruocco et al. [23]; (2) to evaluate their expression upon nutrient starvation. The hypothesis was that the activation/deactivation of specific writers/erasers may occur upon stress. In the current study, we selected the mentioned microalgal species because we have previously exposed these species to stressful culturing conditions, specifically nutrient starvation conditions. These species were also chosen to represent different microalgal classes, enabling the study of potential evolutionary paths leading to gene loss and gain. In addition, they are widespread species and have shown biotechnological interest too, by producing compounds with anti-inflammatory, antiproliferative, and antifungal activities [26,27,28,29,30,31]. In particular, we have also performed RNA sequencing for each of the four mentioned species [26,27,28,29] in both control and nutrient starvation conditions. In the present study, we aim to mine the transcriptomes currently available in public databases in order to identify transcripts coding for the enzymes of interest. We have also selected and designed primers for specific writers and erasers in one of these species to test their expression in various stressful conditions. The comparative transcriptomic results of the current work may be of interest for both the marine ecology and biotechnology communities, because epitranscriptomic regulation may be a crucial step of the activation of enzymatic pathways involved in the synthesis of peculiar compounds involved in microalgal defensive strategies and chemical communication repertoire [32,33,34]. In addition, the metabolites produced can captivate biotechnology researchers interested in bioactive compounds, with possible human applications as well as toxin production monitoring [35,36,37,38]. RNA modifications affect diverse aspects of RNA metabolism, and they are emerging as important players in the post-transcriptional regulation of gene expression, by affecting splicing, nucleus-to-cytoplasm export, and translational ability. The information gained from both DNA and RNA methylation analyses will be very useful for drug discovery research because epigenetics modifiers often regulate the transcription and activation of a particular enzymatic pathway. An example is available for a marine-derived fungus, for which the use of histone deacetylase inhibitors induced the production of bioactive phenolic metabolites [39]. The epitranscriptomics mechanisms (such as m6A RNA methylation) acting in the regulation of gene expression in microalgae and the differences existing in these kinds of regulatory networks between marine microalgae and higher plants is completely unknown so far, and the current study is proposed to start filling this gap.

## 2. Results

### 2.1. New Functional Annotation of the Four Transcriptomes

In order to provide an exhaustive functional annotation and to exploit the biological information obtained thanks to the comparative approach, the encoded proteins from the assembled transcripts of the four species have been predicted. In this way, for each predicted protein, several functional annotations are often available. Remembering that these files contain multiple protein versions of the same assembled transcript, and with the aim of reducing redundancies, we collapsed the protein isoforms, obtaining exactly one protein for each transcript. 

To provide a comparative tool that is able to quantify the biological information content of the analyzed species, highlighting insights on the genome architecture and organization of the compared species, we processed the data coming from the functional annotation procedure. The results of this analysis simultaneously compare the protein domains’ content (InterPro), the protein families’ content (PFAM), and the metabolic information (PATHWAYS) of the four species. All the detected protein domains, together with all the related occurrences detected in each species, are listed in Supplementary Table S1.

### 2.2. Comparative Analysis and Network of Writers and Erasers

To identify the network of orthologs and paralogs with related functions among different species, we performed a comparative analysis between the transcript collections of *Alexandrium tamutum*, *Amphidinium carterae*, *Cylindrotheca closterium*, and *Tetraselmis suecica*. Looking for writer and eraser genes in the four investigated species, we selected query sequences for the enzymatic classes of interest that had already been detected in the work of Ruocco et al. [23]. These queries (Supplementary File S1) are genes encoding for m6A (N6-methyladenosine) writer and eraser proteins from the terrestrial plants *Arabidopsis thaliana* and *Oryza sativa*, and from the marine plants *Zostera marina* and *Cymodocea nodosa*. In particular, the writers for plants and seagrasses were MTA, MTB, FIP37, VIRILIZER, and HAKAI, while the erasers were ALKBH9B and ALKBH10B.

**Figure 1:**
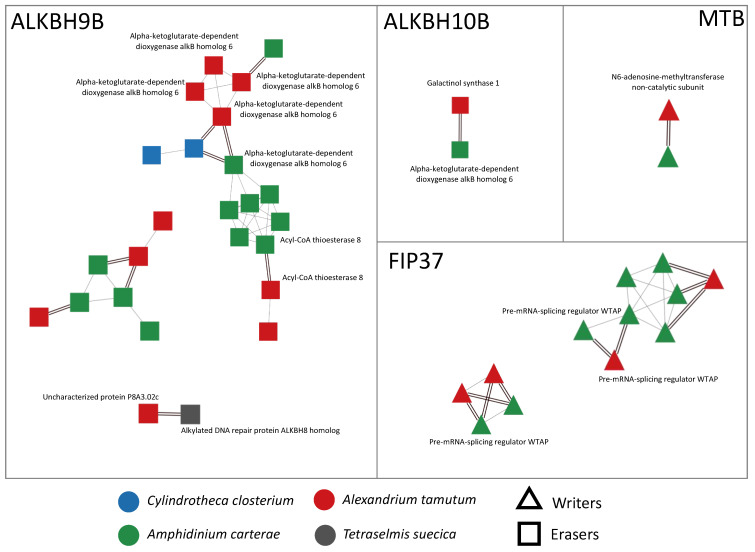
Visualization of networks of orthologs and paralogs related to writers and erasers, obtained using Cytoscape v. 3.10.0. In particular, the writers were MTB and FIP37, while the erasers were ALKBH9B and ALKBH10B. Orthology relationships are represented as double black lines; paralogy relationships are represented as single gray lines. When available, SwissProt functional annotation is shown next to each node.

Writers and erasers that, from now on, we will define as Genes Of Interest (GOIs) of *A. tamutum*, *A. carterae*, *C. closterium*, and *T. suecica* were identified via a comparative approach between their transcript collections and the selected query sequences (Table 1). The IDs of the identified GOIs were subsequently detected in the previously defined networks of orthologs and associated paralogs. All the networks that include the identified GOIs are shown and summarized in Figure 1.

In microalgae, we found almost all the genes encoding for writers and erasers already detected in seagrasses (ALKBH9B, ALKBH10B, MTB, and FIP37) (Figure 1), with the exception of three writers (MTA, VIRILIZER, and HAKAI) that were not present at all in the sequenced transcriptomes. In detail, we detected ALKBH9B in all the considered species, while ALKBH10B, MTB, and FIP37 were exclusively found in the transcriptomes of the dinoflagellates *A. tamutum* and *A. carterae*. Moreover, the protein domain analysis highlighted the presence of some conserved domains, such as the alpha-ketoglutarate-dependent dioxygenase AlkB-like domain (IPR027450) in the ALKBH9B and ALKBH10B networks, the nucleotide-diphospho-sugar transferases domain (IPR029044) in the ALKBH10B network, or the MT-A70-like domain (IPR007757) in the MTB network. The HotDog domain superfamily, which is usually found in thioesterases and thiol ester dehydratase-isomerases, was instead found in *A. tamutum* and *A. carterae* species, and was associated with the ALKBH9B network (Supplementary Table S2).

### 2.3. Genome Searching

Although all the query sequences related to the erasers were detected, it is evident that some of the writer sequences, i.e., MTA, VIRILIZER, and HAKAI, are lacking from the transcript collections of all our species of interest. In addition, ALKBH10B, MTB, and FIP37 are not present in *C. closterium* and *T. suecica*. Since the analyzed transcriptomes depend on the experimental conditions that led to their assembly, we also decided to check the presence of the selected query sequences in the corresponding genomes. This analysis was performed by scanning the corresponding genomes for each species of interest (when publicly available) or those of related species (when not available for the same species). In particular, in the case of *Cylindrotheca closterium* and *Tetraselmis suecica* (for which the genome assemblies are not publicly available), the genomes of strictly related species, i.e., *Cylindrotheca fusiformis* and *Tetraselmis striata*, were considered. The results obtained from the mapping analysis confirmed the presence of all the considered queries in the selected genomes (Table 2).

For these reasons, we concluded that the lack of MTA, VIRILIZER, and HAKAI writer sequences from all the transcriptomes, and of ALKBH10B, MTB, and FIP37 from *C. closterium* and *T. suecica* transcriptomes, appears to be caused more by a lack of their expression in the experimental conditions used to assemble the transcriptomes rather than a real lack of these genes from their related genomes.

### 2.4. Transcriptome Differential Expression Analysis

Differential expression analyses showed that many of the identified transcripts coding for writers and erasers were not differentially expressed in the conditions considered for the RNA sequencing experiments. Nevertheless, two transcripts were differentially expressed. In particular, transcripts vo_c1_Locus_8663_Transcript_1/7_Confidence_0.300_Length_2289 and vo_c3_Locus_6426_Transcript_2/2_Confidence_0.250_Length_1098 coding for the eraser ALKBH9B and the writer FIP37, respectively, were significantly differentially expressed between the control and the nitrogen starvation conditions in which *Amphidinium carterae* was cultured, suggesting their possible involvement in the responses of the microalga-to-nitrogen constrains. In particular, vo_c1_Locus_8663_Transcript_1/7_Confidence_0.300_Length_2289 (ALKBH9B) had a fold change (FC) of 5.31726267 with a *p*-value of 1.88 × 10^−5^ in the nitrogen starvation condition relative to the control, while vo_c3_Locus_6426_Transcript_2/2_Confidence_0.250_Length_1098 (FIP37) showed a FC of −5.1793439 with a *p*-value of 3.33 × 10^−5^.

### 2.5. A. carterae Cultured in Various Stressful Culturing Conditions 

*A. carterae* was also cultured in various stressful conditions (more severe than for the transcriptome sequencing, i.e., nitrogen depletion, phosphate depletion, and two concentrations of Zinc, 5 and 10 times more, with respect to the control) in order to investigate cell growth and differential expression analyses of selected writers and erasers (the writers MTB and FIP37, and the erasers ALKBH9B and ALKBH10B). The results showed that stressful conditions induced early aging (cells entered before in the decline phase) in *A. carterae* cells and that the strongest stress for the cells was represented by the 10 times addition of Zn (named 10 Zn). These results highlighted a net growth rate of 0.45 d^−1^ in the control condition, 0.43 d^−1^ in nitrate depletion, 0.39 d^−1^ in phosphate depletion, 0.44 d^−1^ in 5 times addition of Zn, and 0.22 d^−1^ in the 10 times addition of Zn condition.

### 2.6. Reference Gene Assessment for Reverse Transcription-Quantitative PCR (RT-qPCR) in A. carterae

**Figure 2:**
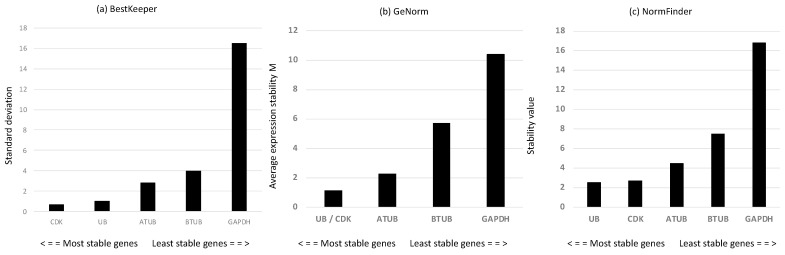
(**a**) Standard deviation values of the derived crossing points for each putative reference gene using BestKeeper analysis [40]; (**b**) gene expression stability M obtained by the stepwise exclusion of more variable genes until the identification of the most stable gene couple performed using geNorm analysis [41]; (**c**) expression stability values determined using NormFinder [42].

Considering that reference genes (RGs) may change depending on the conditions studied, we first screened a panel of putative RGs—alpha and beta tubulins (ATUB and BTUB, respectively), ubiquitin (UB), cyclin-dependent kinase 3 (CDK), and glyceraldehyde 3-phosphate dehydrogenase (GAPDH). According to the mathematical approach of BestKeeper [40] (Figure 2a), the most stable RGs with the lowest standard deviation (SD) were CDK and UB. GeNorm [41] analysis confirmed the results of BestKeeper, showing that the two most stable genes, with the lowest expression stability (M), were UB and CDK (Figure 2b). According to the statistical approach of NormFinder [42], the best reference genes, with the lowest stability values, were UB and CDK, followed by ATUB (Figure 2c). The rank pattern was the same as for Bestkeeper and geNorm analyses. Overall, the results suggested that the best reference gene couple were CDK and UB. Hence, we use them for gene expression investigation of the genes of interest.

### 2.7. RT-qPCR for ALKBH9B, ALKH10B, FIP37, and MTB in A. carterae 

**Figure 3:**
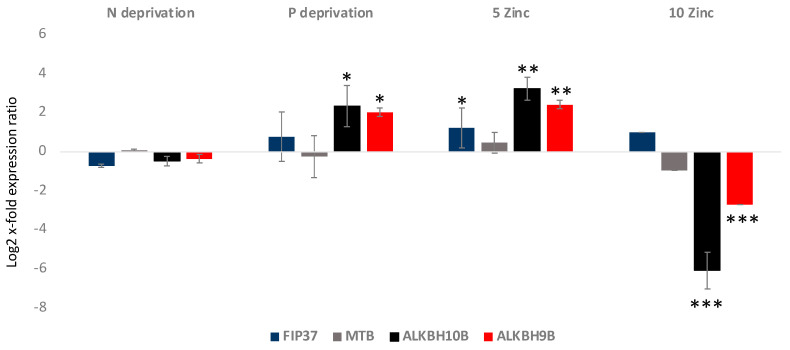
Relative gene expression in *A. carterae* cultivated in nitrogen deprivation (N deprivation), phosphate deprivation (P deprivation), and 5 and 10 times enriched medium with Zn (5 Zinc and 10 Zinc, respectively) with respect to the control medium. The figure shows the writers’ (FIP37 and MTB) and the erasers’ (ALKBH10B and ALKBH9B) gene expression levels in terms of log2 x-fold expression ratio (y-axis, mean ± SD) in *A. carterae* in the various cultivation conditions with respect to the control medium (represented in the figure by the x-axis). Data are normalized with the best RGs—UB and CDK (*n* = 3; * *p* < 0.05, ** *p* < 0.01, *** *p* < 0.001, Student’s *t*-test).

Gene expression analyses showed that FIP37 was up-regulated in the 5 Zinc condition, while it did not show significant variations in the others (*p* < 0.05 for 5 Zinc and *p* > 0.05 for the others) (Figure 3). MTB did not show significant variations in the analyzed culturing conditions (*p* > 0.05 for all). Regarding the erasers, ALKBH10B was significantly 2.5 log2 x-fold and 3.2 log2 x-fold up-regulated in P deprivation and 5 Zinc (*p* < 0.5 and *p* < 0.01, respectively), respectively. In addition, it was −6.1 log2 x-fold significantly down-regulated in the 10 Zinc condition (*p* < 0.001), while it did not show significant variations in the N deprivation condition. ALKBH9B, as was the case with ALKBH10B, was significantly up-regulated in the P deprivation and 5 Zinc conditions, at 2 log2 x-fold and 2.4 log2 x-fold, respectively (*p* < 0.5 and *p* < 0.01, respectively), and −2.7 log2 x-fold down-regulated in the 10 Zinc condition (*p* < 0.001). It did not show significant variations in the N deprivation condition.

## 3. Discussion

For the first time, this study led to the identification in microalgae of transcripts encoding for writers and erasers previously found in higher plants and seagrasses. In particular, the writers MTB and FIP37 and the erasers ALKBH9B and ALKBH10B were found in the microalgal transcriptomes considered in this study, while the three writers MTA, VIRILIZER, and HAKAI were absent. Genome searching analyses also detected them, suggesting that the experimental conditions used for RNA sequencing experiments were not able to activate MTA, VIRILIZER, and HAKAI expression. Overall, writers and erasers previously identified in higher plants and seagrasses were also found for microalgae. However, while ALKBH9B was found in all the considered transcriptomes species, i.e., the diatom *C. closterium*, the green alga *T. suecica*, and the dinoflagellates *A. carterae* and *A. tamutum*, ALKBH10B, MTB, and FIP37 were exclusively detected in the dinoflagellate transcriptomes. These results give new insights into epitranscriptomics in microalgae and their possible involvement in microalgal stress responses. In addition, the differential expression of specific transcripts encoding for the eraser ALKBH9B and the writer FIP37 have been observed when the microalga *A. carterae* has been exposed to nitrogen starvation stressful conditions. ALKBH9B has been shown to remove m6A modification from RNA in vitro in *Arabidopsis thaliana* during viral infection [43], while FIP37 has been identified as a core component of the plant m6A methyltransferase complex responsible for shoot stem cell fate in *Arabidopsis* [17]. Our results highlighted a significant up-regulation of a transcript encoding for ALKBH9B, and an equally significant down-regulation of a transcript encoding for FIP37. Their differential expression was also detected in the circadian rhythm study during a 24 h cycle in the seagrasses *Cymodocea nodosa* and *Zostera marina* [23]. In both marine plants, transcripts related to m6A methylation always peaked during the dark period. We designed primers for FIP37 and MTB writers, and for ALKBH9B and ALKBH10B erasers, and analyzed their expression in severe stressful conditions (nutrient deprivation rather than the nutrient starvation used for transcriptome and metal addition at two different concentrations). The relative gene expression analysis using RT-qPCR showed that expression levels of the writers FIP37 and MTB and the erasers ALKBH10B and ALKBH9B varied depending on the culturing conditions of the microalga *A. carterae*. In particular, ALKBH9B showed the same pattern of ALKBH10B, consistent with their functions. A recent study by D’Aquila et al. [44] also showed that nutrient availability induced a remodeling of writer, reader, and eraser gene expressions in rats. Similarly, Yang and Wang [45] discussed the epitranscriptomic mechanisms of metal toxicity in human cells exposed to various metals, showing that their dysregulations are also involved in cell transformation and tumorigenesis. To our knowledge, our study is the first to show metal exposure effects on epitranscriptome-related genes in marine microalgae and plants in general.

The transcriptomes of *A. carterae* and *A. tamutum* were also particularly redundant in transcripts encoding for the writer protein FIP37 and the eraser protein ALKBH9B. These sequences may be different isoforms and/or transcript variants. The reason for such a redundancy can be partially ascribed to the fact that dinoflagellate genomes are characterized by huge genome sizes (∼3–245 giga bp of DNA held in from several to >100 chromosomes [46,47,48]) and redundancy compared to diatoms and green algae. Dinoflagellates are in fact reported to be characterized by high redundancy and high gene number [46]. However, this redundancy was not observed for the other writers and erasers, and, hence, additional investigations are necessary to shed light on the functional role of these variants.

Finally, comparative transcriptomic studies, like the one reported in the current work, have been applied for both terrestrial and marine species [49,50,51], and can be a great method to reveal the adaptation mechanisms to different environments (e.g., temperate versus tropical or polar) or to different stressful situations (e.g., nutrient starvation and ocean acidification), especially in light of the climate changes expected in the coming years. In fact, various authors have studied transcriptomic changes in microalgae after exposure to stress conditions, such as nutrient starvation, especially for lipid accumulation studies [52,53,54,55].

Epitranscriptomic regulation is a regulatory step for gene expression activation and can have the same activity for microalgae as well. Additional studies in this field may give new insights into microalgal gene expression regulation during daily circadian rhythms, growth phases, approaching nutrient, light, salinity, and other variations they may encounter at sea, as well as upon stress exposure in laboratory conditions. 

Marine organisms are continuously exposed to stress biotic and abiotic constrains, and, when possible, have evolved several adaptation mechanisms in order to cope with stress and survive [56,57,58,59,60,61,62,63,64]. Epitranscriptomic investigations fit well with the increasing consciousness and apprehensiveness about climate change, the necessity to monitor them, to clarify regulatory processes at the gene level, to identify new tools to keep track of stress conditions, and to find eco-sustainable mitigating solutions. Compared to terrestrial organisms, epitranscriptomic studies for marine organisms are less frequent. Some marine epitranscriptomic studies are available for the oyster *Crassostrea gigas* [65], the coral *Acropora hemprichii* [66], and seagrasses *Cymodocea nodosa* and *Zostera marina* [23]. To our knowledge, there are no already published studies on microalgal epitranscriptomics, except a study on tRNA modifications in the picoeukaryote *Micromonas commode* under phosphate starvation. While epitranscriptomic changes have been previously associated with lophotrochozoan development in oysters [65], to circadian rhythms in seagrasses [23], and to heat stress in corals [66], the current study focuses on nutrient starvation/depletion and metal stress exposure. Hence, this work will provide the first report on the epitranscriptomic-related transcripts of writers and erasers in microalgae producing compounds with biotechnological interest. The current work opens new possible challenges for marine ecology, especially for better understanding the molecular mechanisms at the base of microalgal bloom dynamics, community composition, carbon cycling, and large-scale ocean biogeochemistry. This pioneer work related to the epitranscriptomic mechanisms acting in the regulation of gene expression in microalgae could be a subject of great interest for the marine biotechnology community as well, because it represents a new horizon for the possible manipulation of marine bioproduct synthesis. Previous studies in endophytes, for instance, have shown how epigenetic modifiers can influence the production of volatile organic compounds [67]. Similarly, a study on the marine-derived fungus *Penicillium brevicompactum* showed that the use of histone deacetylase inhibitors (i.e., nicotinamide and sodium butyrate) allowed the production of various phenolic compounds [39]. Hence, methylation/demethylation may influence the activation of metabolite biosynthetic pathways, increasing the production of bioactive molecules. The microalgal species selected in the current study have been shown to have antiproliferative [30,31], antioxidant [68], and anti-inflammatory [69] activities. Some of the bioactive compounds responsible for these activities have been identified as amphidinol 22, lysophosphatidylcholines, and pheophorbide a [31,69]. For this reason, going deeper into the understanding of epitranscriptomic regulation will shed light on the molecular mechanisms at the base of bioactive compound synthesis. Considering the huge microalgal biodiversity, additional investigations are necessary for clarifying the presence/absence of writers and erasers in the transcriptome/genome of other microalgal classes and for exploring their functional roles in the regulation of gene expression.

## 4. Materials and Methods

### 4.1. Experimental Design

In this study, four microalgal species have been considered, namely the dinoflagellates *Alexandrium tamutum* and *Amphidinium carterae*, the diatom *Cylindrotheca closterium*, and the green alga *Tetraselmis suecica.* These species were selected for the following reasons: i. they belong to various phyla, ii. stress exposure experiments have been previously performed, and iii. transcriptomes in both control and stressful conditions are available. As described in already published works [26,27,28,29], these species have been cultivated in specific media (Guillard’s F/2 for *C. closterium*, Keller’s medium for *A. carterae* and *A. tamutum*, and Guillard’s F/2 medium without silicic acid for *T. suecica*), depending on the specific nutrient requirements for growth (e.g., silicic acid for diatoms). Similarly, stressful conditions were selected depending on these requirements (e.g., limiting silicic acid for the diatom, which requires it for the construction of the cell wall). In particular, *Alexandrium tamutum* (strain FE107 from the Stazione Zoologica culture collection) was cultured in both control (Keller medium -K [70]) and phosphate starvation conditions (P-starvation experiment, 0.5 μM PO_4_^2−^ rather than 36 μM PO_4_^2−^ of the control condition) [29]. The de novo transcriptome was sequenced, and reads were deposited under the series entry PRJNA632001 in the Sequence Read Archive (SRA) NCBI database [71]. *Amphidinium carterae* (CCMP1314) was also cultured in both control K medium and nitrogen starvation (30 μM of NO^3−^, rather than 883 μM; N starvation condition) [28]. Reads of the transcriptome in both conditions have been deposited in GenBank (GEO database [72]) and are freely available under the series entry GSE94355. The diatom *Cylindrotheca closterium* was cultured in Guillard’s F/2 medium [27,73] and in Si starvation experiments, using 36 μM Si(OH)_4_ rather than 107 μM Si(OH)_4_. De novo transcriptome reads have been deposited and are freely accessible in the NCBI SRA database (PRJNA577416). Finally, the green microalga *Tetraselmis suecica* (CCMP906) was cultured in Guillard’s F/2 medium without silicic acid for the control condition and in the same medium, but 30 μM of NO^3−^ rather than 883 μM, for the N starvation stressful condition [26]. Also in this case, de novo transcriptome reads have been deposited in the GenBank GEO database under the series entry GSE109461. As described in already published works [26,27,28,29], the differential expression analyses were performed with R package DESeq for *A. carterae* and *T. suecica* and EBSeq for *A. tamutum* and *C. closterium* [26,27,28,29,74,75].

### 4.2. Functional Annotation

We mined the already available transcriptomes in public databases for the considered four microalgal species. Protein sequences were predicted from transcripts by using TransDecoder software v 5.3.0. (https://github.com/TransDecoder/TransDecoder/releases, accessed on 18 July 2024). Coding sequences were identified using the software based on the following: (1) a minimum length Open Reading Frame (ORF; 100 by default to minimize the number of false positives); (2) an internal score system; and (3) if a candidate ORF is entirely included within the coordinates of another candidate ORF, the longer one is reported. However, a single transcript can report multiple ORFs if the minimum length is reached. The results of this analysis were the translated proteins of the starting assembled transcriptomes. In many cases, these included more than one predicted protein for each assembled transcript, since a single transcript may result in multiple open reading frames (ORFs) if the minimum length is reached during the translation step.

The functional annotation of the predicted proteins was performed with the software InterProScan (version 5.28), a comprehensive tool that is able to assign functions or signatures by querying 14 different biological databases, including, among others, CDD (Conserved Domain Database), INTERPRO (protein sequence analysis and classification), PATHWAYS (metabolic pathways from KEGG, REACTOME, and METACYC databases), PFAM (collection of protein families), PROSITE PROFILES (protein database using profiles as motif descriptors), SUPERFAMILY (database of structural and functional annotation for all proteins and genomes), and TIGRFAM (curated multiple sequence alignments for protein sequence classification). In the end, the output of these analyses included all the predicted proteins with all the different functional annotation signatures. We automatized this step by selecting for each transcript the corresponding protein with the highest number of functional annotations, filtering out all the other isoforms.

### 4.3. Comparative Analysis

We designed and implemented an automatized pipeline that included the prediction of orthology and paralogy relationships for all the considered species, followed by a network construction step. First of all, sequence similarity searches between the considered transcriptomes were performed using the tBLASTx package of BLAST+ suite version 2.6.0 [76]. The prediction of orthology relationships was performed using Bidirectional Best Hits (BBHs) methodology [77]. The prediction of paralogy relationships was obtained using an automatized procedure that defines an e-value threshold that maximizes the number of networks of duplicated genes, based on the work of Rosenfeld and DeSalle [78]. Finally, the network construction step took as input all the results coming from the orthologs and paralogs prediction, merging them into networks of orthologs and paralogs using COMPARO, an in-house software written in the python programming language. The methodology at the base of COMPARO is described in two previous work [49,79].

### 4.4. Query Sequences Selection and Screening

As queries for our subsequent analyses, we selected genes encoding for m6A (N6-methyladenosine) writer and eraser proteins from the terrestrial plants *Arabidopsis thaliana* and *Oryza sativa*, and from the marine plants *Zostera marina* and *Cymodocea nodosa* (see Supplementary File S1), as identified in a previous work [23]. To detect writers and erasers, (Genes Of Interest, or GOIs), a tBLASTn analysis with the BLAST program [76] was performed by scanning the transcript collections of the four investigated species with the selected query sequences. The IDs of the identified GOIs were subsequently searched and detected in the networks of orthologs and associated paralogs previously detected, as described above. 

### 4.5. Genome Searching

**Figure 4:**
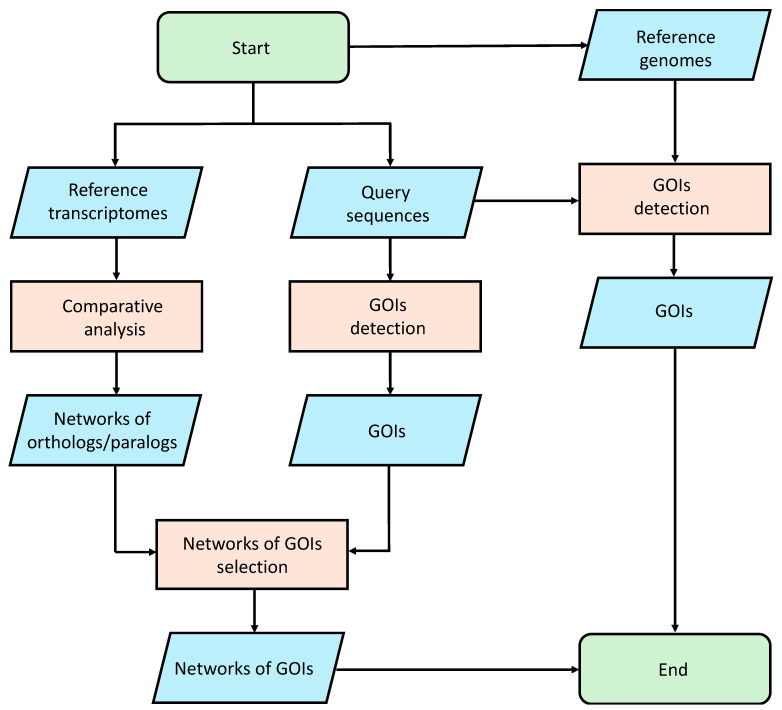
Bioinformatic pipeline used for the identification of Genes Of Interest (GOIs).

We performed an analysis using the gmap [80] tool to align the selected query sequences on the reference genomes. This study made use of publicly available genomic data (see Data Availability Statement). The *Amphidinium carterae* genome is publicly available at Genome at the National Center for Biotechnology Information (NCBI; GCA_019702695.1). Since the *Alexandrium tamutum* genome is not available, we assume that the results obtained from the analysis on the *A. carterae* genome can be translated to *Alexandrium tamutum*, because these two organisms belong to the same phylum. Since the *Cylindrotheca closterium* and *Tetraselmis suecica* genomes are not publicly available, we used as reference to perform the alignment procedure the public genomes of two different organisms of the same genus, i.e., *Cylindrotheca fusiformis* (GCA_019693525.1) and *Tetraselmis striata* (GCA_006384855.1). In order to remove unreliable results, an identity percentage cutoff of 40% was applied to the mapping results. The entire pipeline is summarized in Figure 4.

### 4.6. Microalgal Culturing and Harvesting for Reverse Transcription-Quantitative PCR (RT-qPCR)

*A. carterae* (CCMP1314) was cultivated in Keller medium in 1 L flasks. For the control condition, normal Keller medium was used [70]. Stressful conditions were performed using nitrogen depletion, phosphate depletion, and Zinc addition at two concentrations (5 and 10 times higher, with respect to the control repleted Keller medium) [81,82,83]. Cultures were maintained in a climate chamber at 20 °C on a 12:12 h light–dark cycle at 110 µmol photons m^−2^ s^−1^. Initial cell concentrations were 5000 cells/mL for each experiment, and the culture growth rate was monitored, using the equation for net growth estimates [84].

Culture aliquots in triplicate were collected during the stationary phase and were centrifuged for 15 min at 4 °C at 1900× *g* (Eppendorf, 5810 R). For RNA extraction, the pellets were re-suspended in 500 µL of TRIZOL© (Invitrogen, Carlsbad, CA, USA), incubated for 3 min at 60 °C until completely dissolved, and were kept at −80 °C.

### 4.7. RNA Extraction, Retrotranscription, and RT-qPCR

RNA extraction was performed using glass beads (about 200 mg; Sigma-Aldrich, Milan, Italy) for each 2 mL tube, incubating and mixing tubes for 10 min at 60 °C and maximum speed in the Thermo Shaker BS100 (Biosan, Riga, Latvia), and proceeding according to the TRIZOL© manufacturer’s instructions. RNA quantity, purity, and quality were assessed using the Nano-Drop (ND-1000 UV-Vis spectrophotometer; NanoDrop Technologies, Silverside, Wilmington, NC, USA) and using gel electrophoresis. For cDNA synthesis, 1000 ng/replicate were retrotranscribed using the iScriptTM cDNA Synthesis Kit (BIORAD, Hercules, CA, USA) following the manufacturer’s instructions. Primers used for reference genes are those included in the panel of putative reference genes (i.e., alpha and beta tubulins, ubiquitin, cyclin-dependent kinase 3, and glyceraldehyde 3-phosphate dehydrogenase) in Lauritano et al. 2017 [28]. Primers for writers and erasers were designed using the software Primer3 v. 0.4.0 (http://frodo.wi.mit.edu/primer3/; accessed on 10 April 2024). Primer sequences for Forward (F) and Reverse (R) oligo, respectively, were: ALKBH9-F (CGTGAAACCCTCGTCCTTCA), ALKBH9-R (TCACAGGTTGCACAGGTGTT), ALKBH10-F (CTTGCCAGTTCTGGTTGCAC), ALKBH10-R (ACACGAGGAGCAGGACTTTG), FIP37-F (CGAGCTCCCGATTCTCTTCC), FIP-R (AGAGAGGGACCGGGAAATCA), MTB-F (GCCCAGCCATACTTCCACTT), and MTB-R (CCATAGAACTAGCTCCGCGG). Primers were synthesized commercially by Sigma-Aldrich (Merck KGaA, Darmstadt, Germany). In order to allow assay cross-comparison and to assure equal PCR efficiencies, oligos were designed to amplify cDNA regions within the 100–200 bp range in size, melting temperature of 60 °C, and length of 20 bp. The best RGs were identified using the software BestKeeper [40], geNorm [41], and NormFinder [42]. RT-qPCR experiments were performed in a CFX384 TM Real-Time System (BIORAD) according to 2X Quantitative Master Mix with SYBR^®^ Green low Rox manufacturer’s instructions (Genespin, Milano, Italy). The PCR volume for each sample was 10 μL, with 5 μL of 2X Quantitative Master Mix with SYBR^®^ Green low Rox, 1 μL of cDNA template (dilution used 1:20), and 4 μL of 0.7 pmol/μL for each primer. Experiments were carried out in triplicate and included three negative controls (as in [28]). Gene expression analyses were performed using the REST tool (Relative Expression Software Tool [85]), while statistical analysis used the GraphPad Prim statistic software, V4.00 (GraphPad Software; http://www.graphpad.com/quickcalcs/; accessed on 3 May 2024).

## Figures and Tables

**Table 1:** List of GOI IDs reported for each species. GOIs are grouped based on the different detected writers or erasers.

GENE ID	Species
**Eraser ALKBH9B**	
TR13281-c0_g1_i1	*Alexandrium tamutum*
TR95800-c0_g1_i1	*Alexandrium tamutum*
TR95800-c0_g2_i1	*Alexandrium tamutum*
tri_n_comp20163_c0_seq35	*Amphidinium carterae*
tri_n_comp20163_c0_seq44	*Amphidinium carterae*
tri_n_comp20163_c0_seq46	*Amphidinium carterae*
vo_c3_Locus_29131_Transcript_3/4_Confidence_0.500_Length_4920	*Amphidinium carterae*
TR10254-c0_g1_i1	*Alexandrium tamutum*
SampleTS2_Locus_13766_Transcript_2/4_Confidence_0.400_Length_902	*Tetraselmis suecica*
TR111002-c0_g1_i1	*Alexandrium tamutum*
TR111002-c0_g1_i2	*Alexandrium tamutum*
TR5514-c0_g2_i1	*Alexandrium tamutum*
TR74917-c0_g1_i1	*Alexandrium tamutum*
TR74917-c0_g1_i2	*Alexandrium tamutum*
TR98782-c0_g1_i1	*Alexandrium tamutum*
tri_c_comp46027_c0_seq1	*Amphidinium carterae*
tri_c_comp48811_c0_seq1	*Amphidinium carterae*
vo_c1_Locus_8663_Transcript_1/7_Confidence_0.300_Length_2289	*Amphidinium carterae*
vo_c1_Locus_8663_Transcript_2/7_Confidence_0.600_Length_1953	*Amphidinium carterae*
vo_c1_Locus_8663_Transcript_7/7_Confidence_0.000_Length_2852	*Amphidinium carterae*
vo_c2_Locus_8417_Transcript_1/6_Confidence_0.333_Length_2144	*Amphidinium carterae*
vo_n1_Locus_3421_Transcript_10/10_Confidence_0.100_Length_2245	*Amphidinium carterae*
vo_n1_Locus_3421_Transcript_6/10_Confidence_0.200_Length_2273	*Amphidinium carterae*
TR12427-c0_g1_i1	*Cylindrotheca closterium*
TR12745-c0_g1_i1	*Cylindrotheca closterium*
**Eraser ALKBH10B**	
TR65690-c0_g1_i1	*Alexandrium tamutum*
vo_c2_Locus_19652_Transcript_2/2_Confidence_0.250_Length_1986	*Amphidinium carterae*
**Writer MTB**	
TR59452-c0_g1_i1	*Alexandrium tamutum*
tri_c_comp49311_c0_seq1	*Amphidinium carterae*
**Writer FIP37**	
TR51739-c0_g1_i1	*Alexandrium tamutum*
TR75118-c0_g1_i1	*Alexandrium tamutum*
tri_n_comp42309_c0_seq1	*Amphidinium carterae*
vo_c1_Locus_4428_Transcript_3/4_Confidence_0.879_Length_868	*Amphidinium carterae*
vo_c3_Locus_26772_Transcript_3/6_Confidence_0.529_Length_887	*Amphidinium carterae*
vo_n1_Locus_16_Transcript_73/225_Confidence_0.003_Length_1180	*Amphidinium carterae*
vo_n2_Locus_4713_Transcript_1/2_Confidence_0.333_Length_891	*Amphidinium carterae*
vo_n3_Locus_21951_Transcript_1/2_Confidence_0.985_Length_839	*Amphidinium carterae*
TR126122-c0_g12_i1	*Alexandrium tamutum*
TR126122-c0_g5_i1	*Alexandrium tamutum*
vo_c3_Locus_6426_Transcript_2/2_Confidence_0.250_Length_1098	*Amphidinium carterae*
vo_n2_Locus_6399_Transcript_2/2_Confidence_0.500_Length_1101	*Amphidinium carterae*

**Table 2:** Results of the genome scanning analysis performed by mapping the query sequences on the considered genomes. In particular, (1) the genome of *Amphidinium carterae* was used as a reference genome for *Alexandrium tamutum* and *Amphidinium carterae*; (2) the genome of *Cylindrotheca fusiformis* was used as a reference genome for *Cylindrotheca closterium*; and (3) the genome of *Tetraselmis striata* was used as a reference genome for *Tetraselmis suecica*.

**(1)** ***Amphidinium carterae***		
query_ID	query_length	identity%
AT1G14710.1_ALKBH10B	2429 bp	44.2
AT1G14710.2_ALKBH10B	2513 bp	45.8
AT1G48980.1_ALKBH9B	1830 bp	48.2
AT1G48980.2_ALKBH9B	1818 bp	46.6
AT1G48980.3_ALKBH9B	1832 bp	48.2
AT1G48980.4_ALKBH9B	1745 bp	48.2
AT2G17970.1_ALKBH9B	1797 bp	45.1
AT2G17970.2_ALKBH9B	1981 bp	45.7
AT2G17970.3_ALKBH9B	2119 bp	45.4
AT2G48080.1_ALKBH10B	1519 bp	46.7
AT3G05680.1_VIRILIZER	6833 bp	100
AT3G05680.2_VIRILIZER	6904 bp	100
AT3G54170.1_FIP37	1262 bp	45.7
AT4G02940.1_ALKBH10B	2112 bp	44.4
AT4G09980.1_MTB	3393 bp	43.4
AT4G09980.2_MTB	3342 bp	46.5
AT4G10760.1_MTA	2227 bp	46.9
AT4G36090.1_ALKBH9B	1830 bp	44.8
AT4G36090.2_ALKBH9B	2025 bp	100
AT4G36090.3_ALKBH9B	1707 bp	45.7
AT5G01160.1_HAKAI	1404 bp	46
NP_001042682.1_MTB XM_015763885.2	4254 bp	40.5
NP_001047707.1_MTA XM_015769953.1	2476 bp	45.4
NP_001048963.2_MTB XM_015776811.2	2832 bp	42.9
NP_001049502.1_ALKBH10B XM_015775307.2	2532 bp	100
NP_001056738.1_ALKBH9B XM_015787389.2	2221 bp	46.3
NP_001057630.2_FIP37 XM_015788828.2	1428 bp	42.6
NP_001064055.1_ALKBH10B XM_015759337.2	2380 bp	42.5
NP_001064723.2_MTB XM_015759054.2	3607 bp	100
NP_001064945.2_HAKAI XM_015758095.2	1685 bp	43.3
**(2)** ***Cylindrotheca fusiformis***		
query_ID	query_length	identity%
AT1G14710.1_ALKBH10B	2429 bp	49.8
AT1G14710.2_ALKBH10B	2513 bp	46.3
AT1G48980.1_ALKBH9B	1830 bp	42
AT1G48980.2_ALKBH9B	1818 bp	42.4
AT1G48980.3_ALKBH9B	1832 bp	42
AT1G48980.4_ALKBH9B	1745 bp	44.9
AT2G17970.1_ALKBH9B	1797 bp	49.7
AT2G17970.2_ALKBH9B	1981 bp	45.1
AT2G17970.3_ALKBH9B	2119 bp	47.6
AT2G48080.1_ALKBH10B	1519 bp	42.1
AT3G05680.1_VIRILIZER	6833 bp	100
AT3G05680.2_VIRILIZER	6904 bp	100
AT3G54170.1_FIP37	1262 bp	44.6
AT4G02940.1_ALKBH10B	2112 bp	42
AT4G09980.1_MTB	3393 bp	100
AT4G09980.2_MTB	3342 bp	100
AT4G10760.1_MTA	2227 bp	84.6
AT4G36090.1_ALKBH9B	1830 bp	46.7
AT4G36090.2_ALKBH9B	2025 bp	46.9
AT4G36090.3_ALKBH9B	1707 bp	46.6
AT5G01160.1_HAKAI	1404 bp	43.1
NP_001047707.1_MTA XM_015769953.1	2476 bp	45.6
NP_001049502.1_ALKBH10B XM_015775307.2	2532 bp	40.6
NP_001056738.1_ALKBH9B XM_015787389.2	2221 bp	53
NP_001057630.2_FIP37 XM_015788828.2	1428 bp	40.5
NP_001064055.1_ALKBH10B XM_015759337.2	2380 bp	47
NP_001064723.2_MTB XM_015759054.2	3607 bp	40.4
NP_001064945.2_HAKAI XM_015758095.2	1685 bp	42.5
**(3)** ***Tetraselmis striata***		
query_ID	query_length	identity%
AT1G14710.1_ALKBH10B	2429 bp	40.5
AT1G14710.2_ALKBH10B	2513 bp	40.5
AT1G48980.1_ALKBH9B	1830 bp	43.7
AT1G48980.3_ALKBH9B	1832 bp	43.7
AT1G48980.4_ALKBH9B	1745 bp	43.7
AT2G48080.1_ALKBH10B	1519 bp	44
AT3G05680.1_VIRILIZER	6833 bp	100
AT3G05680.2_VIRILIZER	6904 bp	100
AT3G54170.1_FIP37	1262 bp	42
AT4G10760.1_MTA	2227 bp	42.4
AT4G36090.1_ALKBH9B	1830 bp	46.7
AT4G36090.2_ALKBH9B	2025 bp	46.1
AT4G36090.3_ALKBH9B	1707 bp	46.1
AT5G01160.1_HAKAI	1404 bp	42.6
NP_001042682.1_MTB XM_015763885.2	4254 bp	100
NP_001047707.1_MTA XM_015769953.1	2476 bp	48.8
NP_001048963.2_MTB XM_015776811.2	2832 bp	40.9
NP_001056738.1_ALKBH9B XM_015787389.2	2221 bp	42.9
NP_001057630.2_FIP37 XM_015788828.2	1428 bp	45
NP_001064055.1_ALKBH10B XM_015759337.2	2380 bp	45.9
NP_001064945.2_HAKAI XM_015758095.2	1685 bp	48.3

## Data Availability

This study makes use of publicly available genomic data. The *Amphidinium caterae*, *Cylindrotheca fusiformis*, and *Tetraselmis striata* genomes are accessible at the Genome partition of the National Center for Biotechnology Information (NCBI) (https://www.ncbi.nlm.nih.gov/genome/?term=Amphidinium+carterae, https://www.ncbi.nlm.nih.gov/genome/?term=Cylindrotheca+fusiformis and https://www.ncbi.nlm.nih.gov/genome/?term=Tetraselmis+striata, respectively, accessed on 18 July 2024). Transcriptomics resources are available at the SRA partition of NCBI database, under the entries PRJNA632001 and PRJNA577416 for *A. tamutum* and *A. carterae*, respectively, and at the GenBank Geo Database under the entries of GSE94355 and GSE109461 for *C. closterium* and *T. suecica*, respectively.

## Supplementary Materials

The following supporting information can be downloaded at: https://www.mdpi.com/article/10.3390/ijms25158005/s1.
